# Convective forces increase CXCR4-dependent glioblastoma cell invasion in GL261 murine model

**DOI:** 10.1038/s41598-018-35141-9

**Published:** 2018-11-19

**Authors:** R. Chase Cornelison, Caroline E. Brennan, Kathryn M. Kingsmore, Jennifer M. Munson

**Affiliations:** 1 Department of Biomedical Engineering and Mechanics, Virginia Polytechnic Institute and State University, Blacksburg, VA 24061 USA; 2 Department of Biomedical Engineering, University of Virginia, Charlottesville, VA 22908 USA

## Abstract

Glioblastoma is the most common and malignant form of brain cancer. Its invasive nature limits treatment efficacy and promotes inevitable recurrence. Previous *in vitro* studies showed that interstitial fluid flow, a factor characteristically increased in cancer, increases glioma cell invasion through CXCR4-CXCL12 signaling. It is currently unknown if these effects translate *in vivo*. We used the therapeutic technique of convection enhanced delivery (CED) to test if convective flow alters glioma invasion in a syngeneic GL261 mouse model of glioblastoma. The GL261 cell line was flow responsive *in vitro*, dependent upon CXCR4 and CXCL12. Additionally, transplanting GL261 intracranially increased the populations of CXCR4^+^ and double positive cells versus 3D culture. We showed that inducing convective flow within implanted tumors indeed increased invasion over untreated controls, and administering the CXCR4 antagonist AMD3100 (5 mg/kg) effectively eliminated this response. These data confirm that glioma invasion is stimulated by convective flow *in vivo* and depends on CXCR4 signaling. We also showed that expression of CXCR4 and CXCL12 is increased in patients having received standard therapy, when CED might be elected. Hence, targeting flow-stimulated invasion may prove beneficial as a second line of therapy, particularly in patients chosen to receive treatment by convection enhanced delivery.

## Introduction

Glioblastoma (GBM) is the most aggressive form of brain cancer and is characterized by invasion into the surrounding brain or parenchyma^1,2^. This invasiveness causes diffuse borders between the tumor and parenchyma, preventing effective resection of all malignant cells. Additionally, because tumor cells that have invaded into the surrounding healthy tissue are increasingly resistant to radiation and chemotherapy, GBM always recurs^3,4^. Therefore, understanding and targeting molecules that regulate glioma cell invasion has therapeutic implications in the treatment of GBM. One signaling axis known to regulate GBM invasion is the CXCR4-CXCL12 pathway. While a potent driver of GBM invasion in static conditions, CXCR4- and CXCL12-mediated invasion in GBM can be enhanced by interstitial fluid flow through a mechanism known as autologous chemotaxis^5–7^. Interstitial flow is the movement of fluid from the vasculature throughout the interstitial tissue space toward draining lymphatics or clearance pathways. This process normally maintains tissue homeostasis, but the leaky nascent vasculature and increased waste production in solid cancers can dramatically increase interstitial pressure and, in turn, interstitial flow^1,8^.

We previously showed that rat and human GBM cell lines respond to flow *in vitro* by increasing invasion^6,7^. Furthermore, regions of high flow (identified by arterial extravasation of Evans blue) correlated with regions of invasion for cell lines as well as patient-derived glioma stem cells^6,7^. *In vitro*, flow-stimulated invasion was mitigated by both blocking the receptor CXCR4 as well as saturating the ligand CXCL12, suggesting this chemokine-receptor pathway plays a key role in glioma cell flow response. It remains unknown, however, if interstitial flow directly stimulates cancer cell invasion *in vivo* and if CXCR4 signaling is similarly implicated. Answering these questions requires a technique to induce convective forces within the tumor *in situ* at a time when heightened interstitial flow may not be fully established on its own.

Convection enhanced delivery (CED) is an experimental technique used in the clinic to overcome high intra-tumoral pressure and increase drug distribution via local infusion^9,10^. A blunt needle is placed into the center of the tumor, and a drug-laden solution is infused to enhance drug transport. In essence, CED drives convective flow through the interstitial spaces in the tumor, mimicking interstitial fluid flow. We used CED in an orthotopic, murine model of GBM to test the hypothesis that convective flow directly stimulates cancer cell invasion *in vivo* and examine the dependence of this response on CXCR4 signaling.

## Results

### GL261 exhibit flow-stimulated invasion *in vitro* in a CXCR4-dependent manner

**Figure 1:**
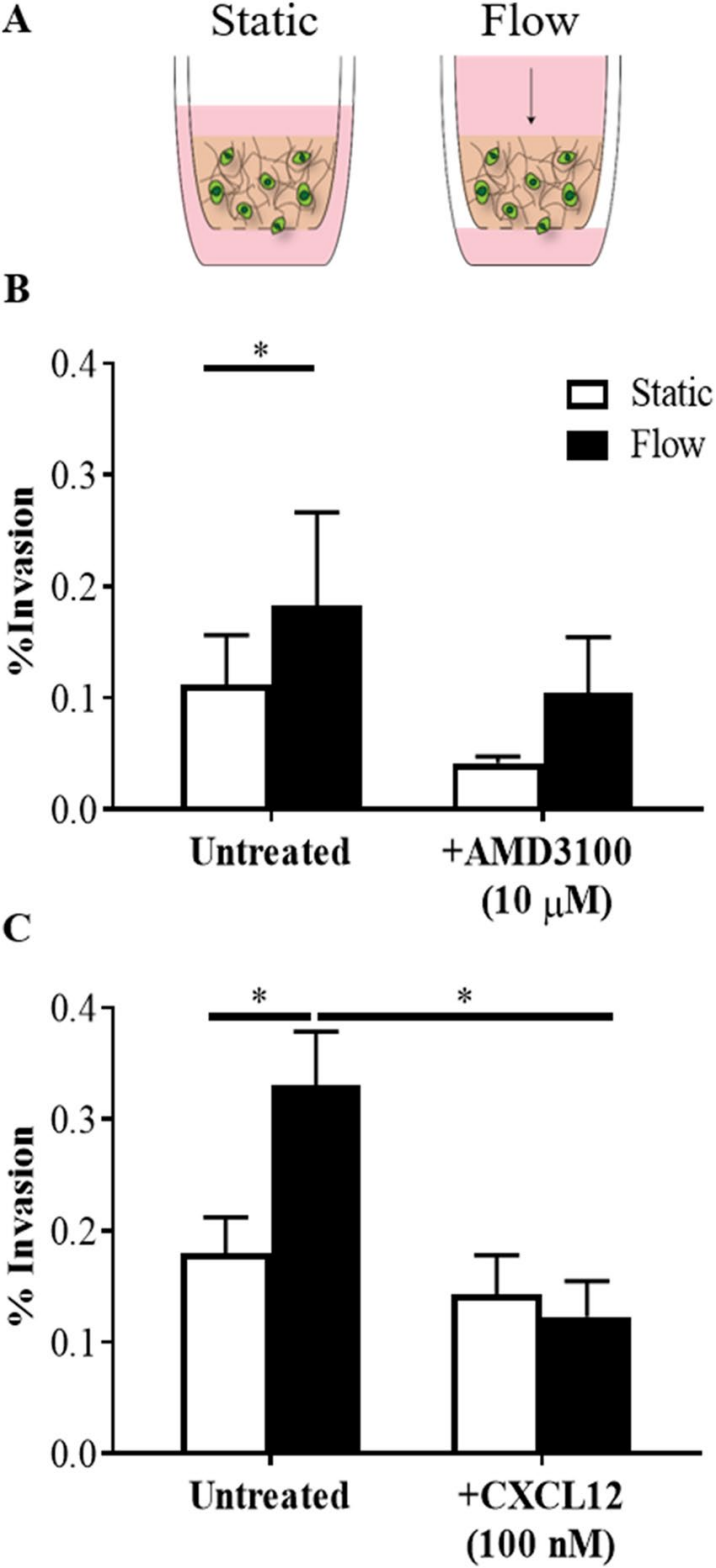
Interstitial flow increases GL261 invasion in a CXCR4-CXCL12 dependent manner. (**A**) Schematic representation of tissue culture insert setup for static and flow experimental conditions. (**B**) Percent invasion of GL261 in static and flow conditions with and without addition of 10 µM AMD3100 (n = 5, *p < 0.05). (**C**) Percent GL261 invasion in static and flow conditions with and without addition of 100 nM CXCL12 (n = 4, *p < 0.05). Bars show standard error.

Prior to *in vivo* assessment, the flow response of GL261 cells was examined *in vitro* using a 3D tissue culture insert model (Fig. 1A)^6^. Under static conditions, 0.1–0.2% of GL261 invaded beyond the semi-permeable membrane (Fig. 1B). The addition of gravity-driven flow significantly increased the percent of cells invading by approximately 1.6 fold (t(4) = 5.931, n = 5, p < 0.01). This flow-stimulated increase in invasion could be mitigated by blocking CXCR4 using 10 µM AMD3100, a small molecule inhibitor of CXCR4 (t(4) = 2.722, n = 5, p > 0.1). Similar results were observed for saturating the cultures (in the gel and on both sides of the tissue culture insert) with 100 nM CXCL12 to eliminate cytokine gradient formation under flow. Ligand saturation significantly decreased the effects of flow (t(4) = 3.545, n = 5, p < 0.05) (Fig. 1C), returning invasion to static levels (t(3) = 2.293, n = 4, p > 0.1). Hence, the flow response of GL261 aligns with the previously proposed mechanism of CXCR4-CXCL12 autologous chemotaxis^1^.

### CXCR4^+^ and CXCR4^+^CXCL12^+^ populations are enriched within *in vivo* tumor samples

**Figure 2:**
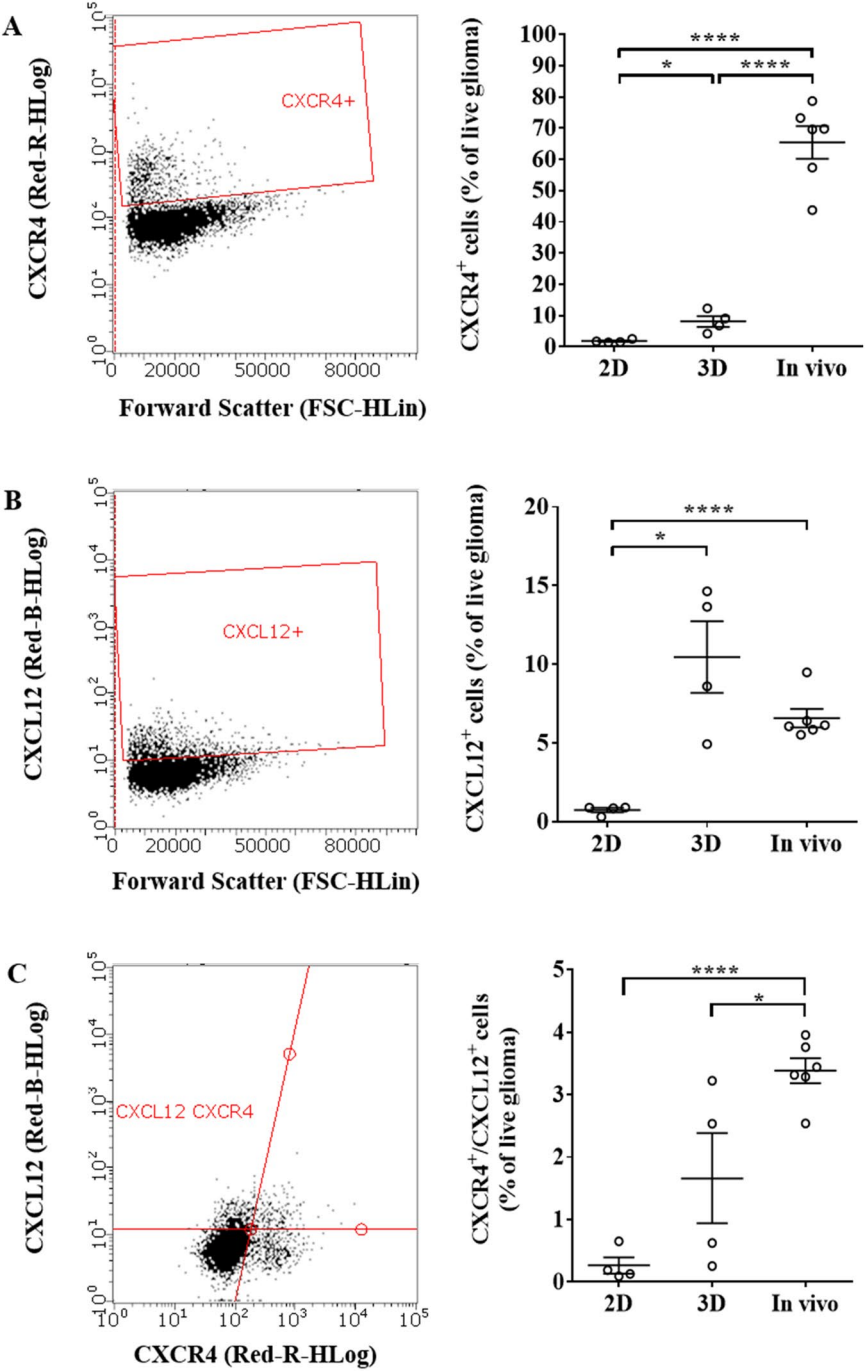
Population-level expression of CXCR4 and CXCL12 in GL261 depends on growth conditions. Flow cytometry was used to determine the percent of CXCR4^+^, CXCL12^+^, and double positive GL261 in 2D, 3D, and *in vivo* environments. Representative plots gated on live glioma cells are shown in the left column for (**A**) CXCR4^+^, (**B**) CXCL12^+^, and (**C**) double positive populations. Correlating quantifications are shown on the right. *p < 0.05, ****p < 0.0001. Bars show standard error.

Because the significance of targeting autologous chemotaxis and flow-stimulated invasion may be influenced by expression levels, we used flow cytometry to characterize GL261 expression of CXCR4 and CXCL12 in different environments. The dimensionality of culture significantly impacted receptor and ligand expression. In 2D, few cells expressed the receptor, ligand, or both (Fig. 2). Embedding the cells in 3D hydrogels significantly increased the number of CXCR4^+^ cells to 8.13 ± 1.71% compared to 1.83 ± 0.25% in 2D culture (t(3) = 3.389, n = 4, p < 0.05) (Fig. 2A). Similar effects were observed on the CXCL12 population (t(3) = 4.14, n = 4, p < 0.05) (Fig. 2B). While there was no difference in the percentage of CXCR4^+^CXCL12^+^ cells between 2D and 3D *in vitro* culture (Fig. 2C), this double positive population increased from 1.66 ± 0.72% in 3D to 3.38 ± 0.49% of total cells *in vivo* (t(8) = 2.767, n = 6 *in vivo* and n = 4 *in vitro*, p < 0.05 compared to 3D). These effects were further amplified for CXCR4 single expression, dramatically increasing from 8.13 ± 1.71% in 3D to 65.4 ± 5.19% *in vivo* (t(8) = 8.653, n = 6 *in vivo* and n = 4 *in vitro*, p < 0.0001 compared to 3D). Expression of CXCL12 *in vivo* was similar to that in 3D culture. Given the role of this receptor/ligand pair on flow response, an enrichment in CXCR4^+^ and CXCR4^+^CXCL12^+^ populations may increase the potential for flow-stimulated invasion *in vivo*.

### Glioma invasion *in vivo* is enhanced by convective flow

**Figure 3:**
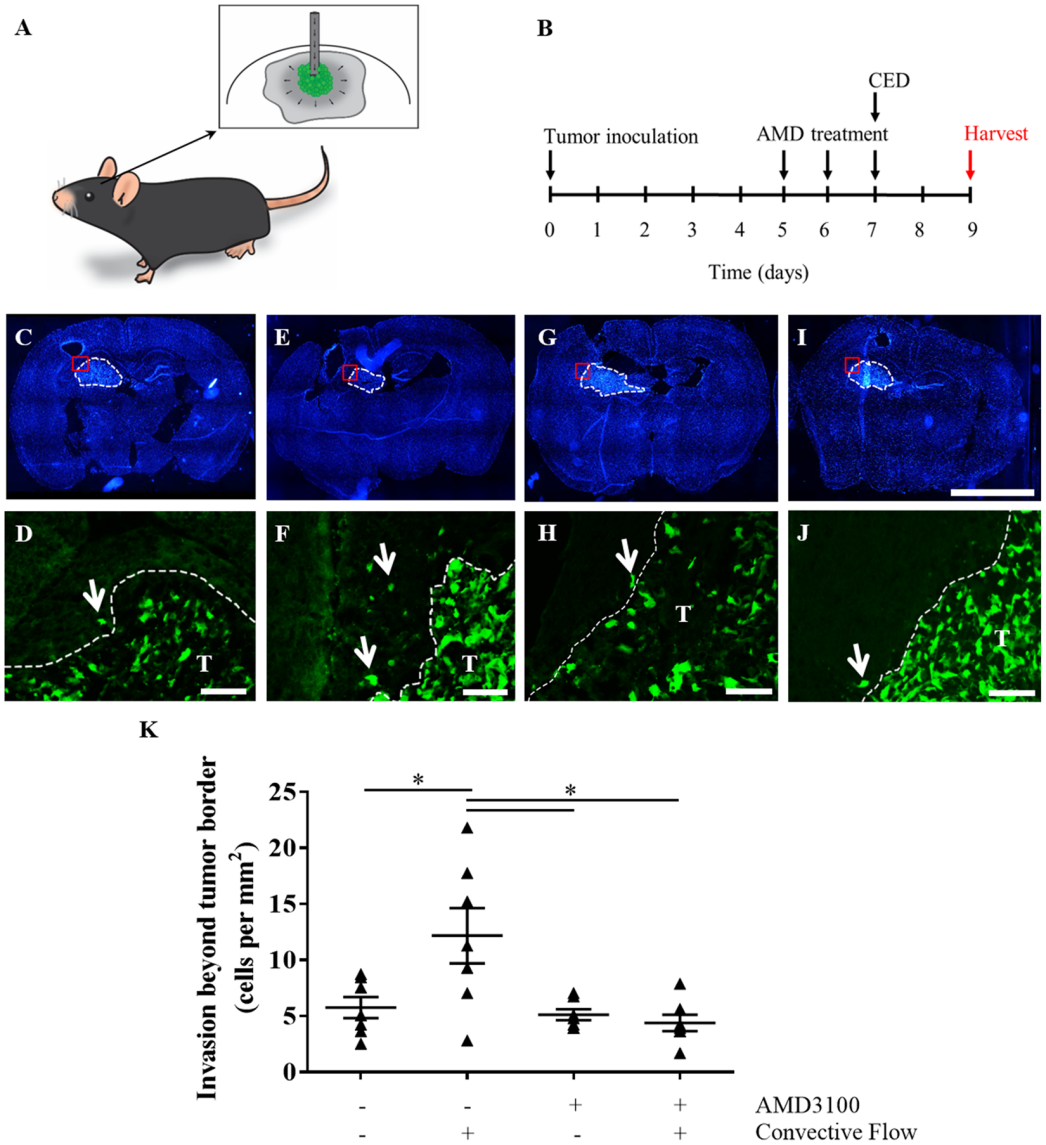
Interstitial flow increases murine glioma cell invasion *in vivo* in a CXCR4-dependent manner. (**A**) Schematic of intratumoral convection enhanced delivery. (**B**) Experimental timeline. (**C**–**J**) Representative fluorescence images of *in vivo* glioma invasion for (**C**,**D**) untreated controls, (**E**,**F**) CED alone group, (**G**,**H**) AMD alone group, and (**I**–**J**) + CED/ + AMD group. Top: Full brain slice scans with nuclei labeled with DAPI (blue), with tumor defined by white dotted line. Scale bar = 1 mm. Bottom: GFP-labeled GL261 tumor cells at the border location depicted above (red boxes). Scale bar = 100 µm. (**K**) Quantification of tumor cells beyond the tumor border averaged per mouse from five locations in three sections through tumors. Bars show standard error.

We examined the effects of convective forces on glioma cell invasion *in vivo* using the therapeutic technique of convection enhanced delivery (CED). A cartoon of the process is shown in Fig. 3A, and an experimental timeline in Fig. 3B. First, magnetic resonance imaging was used to verify the ability to induce fluid convection using CED. A gadolinium contrast agent conjugated to albumin (Galbumin, 25 mg/mL) was infused into the tumors at day 7 at a rate of 1 µL/min. Immediately following CED, the mice were transferred to a 7 Tesla MRI machine to visualize changes in galbumin distribution over time. T2-weighted images were used to identify the location of the tumors (Suppl. Fig. 2A). Using T1-weighted imaging, the signal intensity of intra-tumoral galbumin was observed to change over a 30 minute period, indicative of contrast agent flux (Suppl. Fig. 2B). Five representative slices are shown for one mouse.

Following verification that CED does induce convective flow, a second cohort of mice was used to examine invasion. Convective flow was again induced seven days after tumor inoculation at 1 µL/min, and invasion was assessed two days later using immunohistochemistry. Representative images are shown in Fig. 3C–J, with invasion quantification summarized in Fig. 3K. Untreated (static) tumors had approximately 5.75 ± 0.938 cells/mm^2^ invaded beyond the tumor border into the surrounding tissue. Following CED, the number of invading cells significantly increased to 12.2 ± 2.4 cells/mm^2^ (Fig. 3E,F,K) (t(12) = 2.433, n = 7, p < 0.05). This greater than 2-fold increase to invasion *in vivo* was even more pronounced than the *in vitro* results (1.6-fold change under flow).

### Effects of flow *in vivo* are mediated through CXCR4

**Figure 4:**
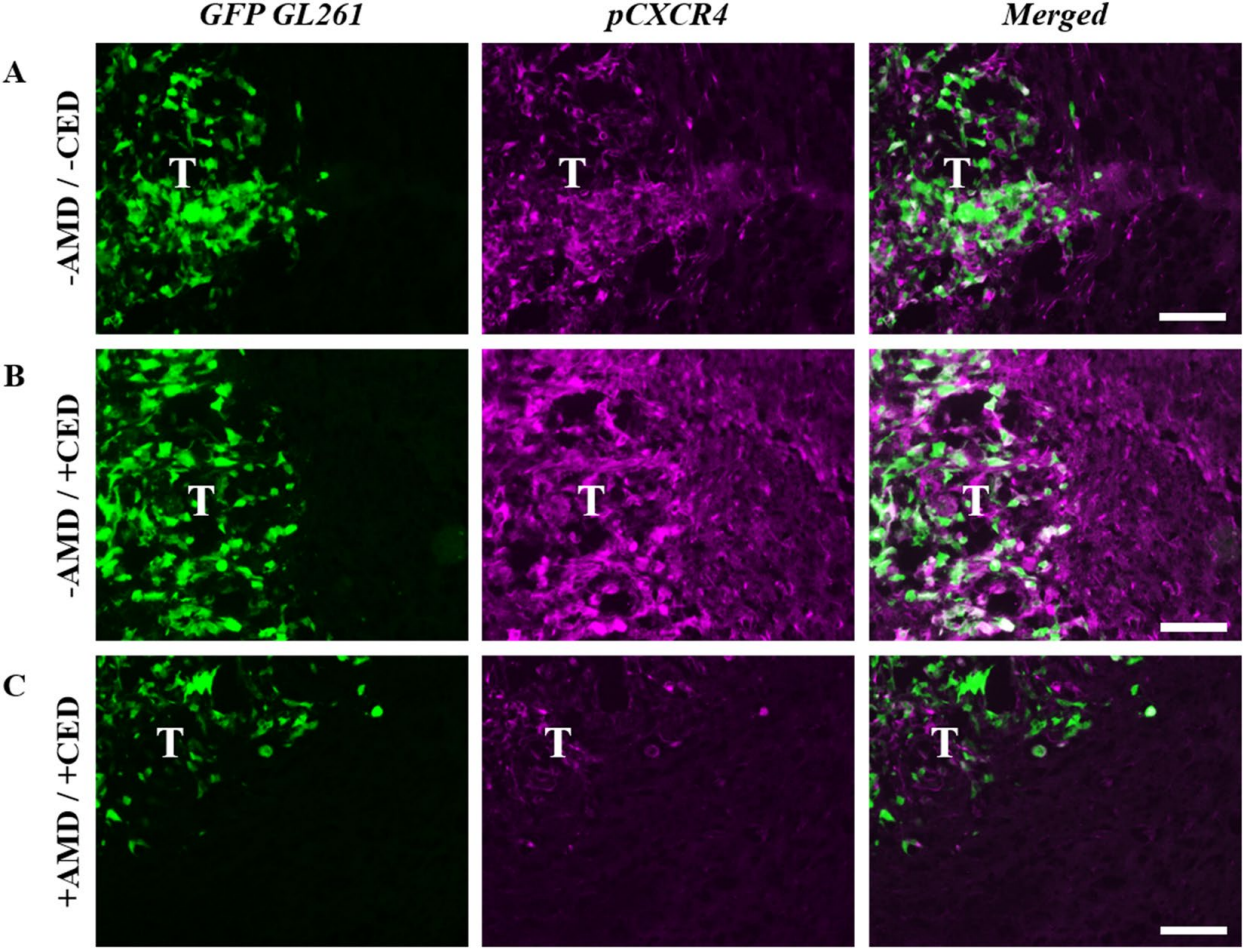
Treatment of GL261 with AMD3100 decreases convection-driven increases in pCXCR4. Representative fluorescence images at GL261 tumor (T) borders of GFP-GL261 (green) and pCXCR4 (magenta) in (**A**) untreated animals, (**B**) animals receiving only CED, and (**C**) animals dosed with AMD3100 for two days prior to CED. Scale bars = 100 μm.

Given the ability of CXCR4 antagonism to reduce flow-stimulated invasion *in vitro*, we examined the effects of administering the CXCR4 antagonist AMD3100 (5 mg/kg) systemically with and without CED^11^. This drug has been delivered to *in vivo* glioma models previously and shows some clinical potential as a secondary therapy^12–14^. In the absence of convective flow (Fig. 3G,H,K), AMD3100 did not significantly alter glioma cell invasion compared to untreated controls at 5.12 ± 0.490 cells/mm^2^ (t(12) = 0.6008, n = 7, p > 0.1). However, treating mice with AMD3100 (Fig. 3I,J,K) significantly reduced the effects of CED on invasion compared to CED alone (t(12) = 3.026, n = 7, p < 0.05). This treatment regimen effectively maintained the number of cells invading beyond the tumor border to 4.38 ± 0.731 cells/mm^2^, not significantly different from that of untreated, static controls. Hence, dosing with AMD3100 prior to convection was able to mitigate flow-stimulated increases to glioma cell invasion. This decrease in flow-stimulated invasion with AMD3100 treatment was also associated with a decrease in CXCR4 phosphorylation, an indicator of receptor stimulation and signaling^6^. Untreated tumors exhibited moderate immunoreactivity for phosphorylated CXCR4 (Fig. 4A), indicating that this signaling pathway is basally active within GL261 tumors *in vivo*. Applying flow via CED markedly increased pCXCR4 immunoreactivity *in vivo* (Fig. 4B), consistent with prior *in vitro* results^6^. Administering AMD3100 prior to CED effectively attenuated increased pCXCR4 staining, observably decreasing immunoreactivity below that of untreated controls (Fig. 4C). Hence, interstitial flow is indeed able to stimulate invasion of glioma cells *in vivo* mediated at least in part through CXCR4 signaling.

### CXCR4 and CXCL12 are increased in patient samples obtained after radiation and chemotherapy

**Figure 5:**
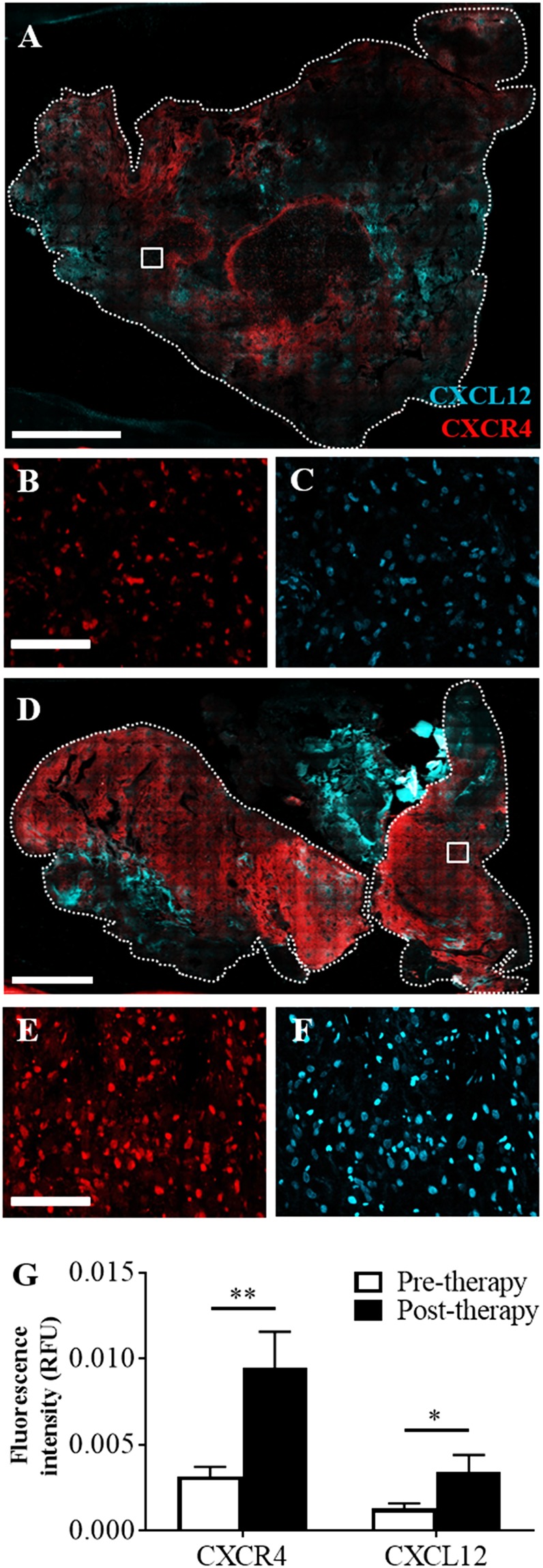
Immunoreactivity for CXCR4 and CXCL12 is increased in patients who received therapy. (**A**) Representative fluorescence image of a resected tumor from a patient prior to therapy stained for CXCR4 (red) and its ligand CXCL12 (cyan). (**B**) Close up of white boxed area for CXCR4, and (**C**) Close up for CXCL12. (**D**) Representative fluorescence image of a resected tumor from a patient after standard of care therapy stained for CXCR4 (red) and its ligand CXCL12 (cyan). (**E**) Close up of white boxed area for CXCR4, and (**F**) Close up for CXCL12. The entire tissue sample is outlined with a white dashed line. Scale bars are 7 mm for A/D and 200 μm for B/C/E/F. (**G**) Quantification of fluorescence intensity from high magnification images (B/C/E/F) at five random locations throughout the tumor sample. *p < 0.05 and **p < 0.01.

Convection enhanced delivery is experimentally used in the clinic to deliver a secondary therapy, meaning this technique is implemented after standard radiation therapy and chemotherapy. In previous publications, it was shown that treatment with radiation therapy, a common therapeutic intervention against glioblastoma, led to increases in flow-stimulated invasion *in vitro*^7^. It was postulated that this effect was due to increases in both CXCR4 and CXCL12 in glioma cells. It is therefore important to consider how standard therapy, including both radiation therapy and chemotherapy, may affect the propensity for flow-stimulated cancer cell invasion. We used immunofluorescence staining to quantify expression of CXCR4 and CXCL12 in tissue samples from patients diagnosed with glioblastoma, with one cohort comprising samples taken prior to therapy and one with samples taken after therapy (Fig. 5). Statistics for the entire and subdivided patient cohorts are summarized in Table 1. Samples were analyzed within the tumor regions of resected samples. Pre-therapy samples showed modest expression of both CXCR4 and CXCL12 (Fig. 5A–C) while samples obtained after therapy exhibited a marked increase in the staining intensity for both markers (Fig. 5D–F). This effect was quantified using image analysis and integrated density measurements (Fig. 5G), confirming that post-therapy samples had significantly increased fluorescence intensity for both CXCR4 (p < 0.01) and CXCL12 (p < 0.05) compared to pre-therapy samples (df = 14; n = 9 pre-therapy and n = 7 post-therapy).

**Table 1:** Patient cohort statistics for samples used to quantify CXCR4 and CXCL12 pre- and post-therapy.

	*Data size*	*Mean age at diagnosis (years)*	*Mean survival*, *(months) if known*
Entire cohort	17	57.00 ± 3.32	15.57 ± 3.74 (n = 7)
*Males*	9	53.11 ± 5.69	10.50 ± 2.50 (n = 2)
*Females*	8	62.67 ± 2.77	17.60 ± 5.02 (n = 5)
Pre-therapy	10	57.60 ± 5.30	11.25 ± 5.04 (n = 4)
Post-therapy	7	56.14 ± 7.35	21.33 ± 4.26 (n = 3)

One pre-therapy sample in each analysis was found to be a statistical outlier by Grubb’s test and was omitted from the analyses. Data are shown as mean ± standard error.

## Discussion

Interstitial fluid flow is a key component of normal physiology; however, emerging evidence suggests that this biomechanical force may also contribute to cancer malignancy. The phenomenon is studied most extensively in breast cancer, where interstitial flow influences both the direction and magnitude of cancer cell migration and promotes activation of, and matrix remodeling by, relevant stromal cells^5,15–18^. Regarding GBM, paths of brain tumor dissemination correlate with bulk fluid pathways^19^. Additionally, we now know that interstitial fluid flow pathways within GBM in mice are complex and heterogeneous, indicating a need for greater understanding of the impacts on cellular invasion^20^. Only recently we showed that interstitial flow indeed increases invasion of both murine and human glioma cells *in vitro* through the chemokine receptor-ligand pair CXCR4-CXCL12^6,7^. Nonetheless, the causal effects of flow on cancer cell invasion have only been studied through *in vitro* experiments, and previous *in vivo* data simply showed correlation. The goal of the current study was to determine if interstitial fluid flow directly stimulates glioma cell invasion in the brain.

The therapeutic technique of convection-enhanced delivery (CED) was used to induce convective flow within brain tumors *in situ*, as evidenced by rapid elimination of an infused contrast agent. CED is a catheter-based method used to by-pass suspected transport limitations between the vasculature and the high-pressure tumor bulk. This technique has been used experimentally and tested clinically for enhancing local perfusion of chemotherapeutics or other drugs in the treatment of GBM^10,21–23^. Here, we found that applying CED at 1 µL/min – the lower end of the clinically-relevant range (1–5 µL/min)^9^ – significantly increased GL261 cell invasion compared to untreated controls at two days after flow application. This greater than 2-fold differential in invasion was more pronounced than in comparative *in vitro* experiments, suggesting an enhanced contribution of flow-stimulated invasion *in vivo*. Because flow response can be mediated through the receptor CXCR4 *in vitro*, and the percentage of CXCR4^+^ GL261 cells dramatically increased upon implantation (8.13 ± 1.71% in 3D vs. 65.4 ± 5.19% *in vivo*; p < 0.0001), we investigated the involvement of CXCR4 signaling on *in vivo* flow-mediation invasion.

Even in the absence of applying CED, we noted moderate immunoreactivity in the tumor bulk for phosphorylated CXCR4, a known marker of CXCR4 activation and signaling. Phosphorylation of CXCR4 occurs primarily due to binding with its ligand, CXCL12, and can lead to myriad signaling events^24^. Convective flow increased pCXCR4 immunoreactivity both in the tumor and the surrounding brain tissue, suggesting the technique of CED may increase chemokine signaling throughout the tumor microenvironment. CXCR4 activation leads to tyrosine-mediated phosphorylation of downstream proteins, including focal adhesion kinases (FAK), and subsequently increased migration^25,26^. Interstitial flow has been shown to localize pFAK in the flow direction, indicative of directional migration or cellular chemotaxis^5,6^. There may be further implications of increased CXCR4 phosphorylation in the surrounding brain since activation of CXCR4 in glia can lead to increased neurotoxicity and pro-tumor phenotypes^27,28^. Additional studies are required to examine if the negative implications of CED (increased invasion and CXCR4 phosphorylation) are counter-balanced by the cytotoxic effects of an infused drug.

Because glioma cells themselves can express CXCL12, it has been proposed that one way interstitial flow stimulates invasion is through a mechanism termed “autologous chemotaxis”^1,5,6,29^. Essentially, *in vitro* and *in silico* experiments suggest that fluid flow creates an anisotropic ligand gradient around individual cells, pushing the chemokine downstream of the cell where it binds to the extracellular matrix and stimulates migration in the direction of flow. We previously showed using an agent based model that heterogeneous populations of glioma cells are exquisitely sensitive to small changes in CXCR4 and CXCL12 co-expressing cells, leading to enhanced flow response via this autologous chemotaxis mechanism^7^. While this previous analysis used expression data from cells *in vitro*, here we found a drastic increase in the populations of CXCR4^+^ and double positive cells upon transplantation, further increasing the implications of flow-stimulated invasion *in vivo*. Although CXCL12 expression did not vary significantly between 3D culture and *in vivo* (and has been shown not to vary in response to flow^6^), CXCL12 is produced by other cells, such as endothelial cells and astrocytes, and is also present in the blood^30–32^. Therefore, ligand availability likely increases upon implantation independent of tumor cell expression.

Using the small molecule antagonist AMD3100, we showed that CXCR4 signaling is likely a primary mechanism by which the GL261 cell line responds to flow. Nonetheless, patient-derived glioma stem cells display heterogeneity in their dependence on CXCR4 versus other receptor-mediated responses to flow such as CD44 mechanotransduction^7^. Thus, supplementation with other targeted therapies delivered via CED may be advantageous. Inhibition of either CD44 or integrin receptors also limits glioma invasion *in vivo* and *in vitro*^33–36^. Interestingly, both of these mechanoreceptors are linked with CXCR4 as co-receptors, acting to either increase affinity to CXCL12 or mediate downstream signaling events^37–39^. While the antagonist AMD3100 specifically inhibits activation of CXCR4 by displacing binding with the N-terminal of CXCL12^40,41^, the binding pocket of CXCL12 overlaps with that of some CXCR4 co-receptors^42,43^. Therefore, AMD3100 may also act to inhibit these other mechanisms. Blocking CXCR4 did not eliminate invasion entirely under static or flow conditions, however. Cancer cell invasion is a multifaceted process regulated by many mechanisms, as previously reviewed by Sayegh *et al*.^44^. Thus, mechanisms other than CXCR4-mediated flow response may concurrently enhance infiltration into the brain such that targeting of many pathways may be required to eliminate invasion.

While not examined here, it is important to consider that CED is most often used experimentally after standard radiation and chemotherapy and in the presence of the tumor bulk. Previous work demonstrated that radiation induces tumor invasiveness by increasing tumor-derived CXCL12 at the invasive tumor border, which may enhance the potential for CXCR4 signaling^45^. Furthermore, irradiation of GL261 cells increases CXCR4 expression in a dose-dependent manner^46^. Using immunofluorescence staining of patient samples, we observed that tissue from patients who had received therapy showed increased immunoreactivity for both CXCR4 and CXCL12 compared to samples obtained from patients prior to therapy. These observations suggest that the increased expression found in mouse cells after therapy may also hold true in humans. Furthermore, CXCR4 is also a purported marker of glioma stem cells^47^ and so increases to CXCR4 expression due to radiation may not only increase the potential for flow-stimulated invasion but also increase malignancy via cancer stem cell expansion.

Ultimately, GBM therapies and delivery strategies have the potential to manipulate both cellular flow response and interstitial fluid pathways and velocities in GBM. The idea that therapy may alter GBM response – while not a new idea – has never been explored in the context of interstitial flow regulation. Therapeutic agents such as bevacizumab and dexamethasone, which act to normalize blood vessels and reduce inflammation, and delivery regimes such as CED could either increase or decrease interstitial flow due to alterations in intratumoral pressure^48,49^. While CED is not a poor choice for drug delivery in GBM, our data imply that therapeutic use of CED may benefit from supplementation with CXCR4 blockade to prevent undesirable consequences on cancer cell invasion. This work offers a first perspective on the unstudied effects of flow-altering delivery strategies and their potential to simultaneously treat and worsen GBM in the absence of inhibiting flow-stimulated mechanisms of invasion.

## Materials and Methods

### *In vitro* invasion assays

GL261 invasion was assessed *in vitro* using 12-well (Millipore PI8P01250) or 96-well (Corning 3374) tissue culture inserts^1^. Cancer cells were seeded at 1 × 10^6^ cells/mL in 3D hydrogels comprising 1.5% rat tail collagen (Corning 354236), 0.2% thiolated hyaluronic acid (Glycosil®; ESI Bio GS220), and 0.1% PEGDA (ESI Bio GS3006). After 20 minutes of gelation, 15 μL of fresh medium was applied on top of the gels. Flow was initiated three hours later using serum-free medium, and cultures were maintained overnight. AMD3100 was used at 10 μM (Sigma A5602) to block the receptor CXCR4 or an excess of 100 nM CXCL12 (Peprotech 300–28 A) was added to prevent chemokine gradient formation. The membranes were then fixed in 4% paraformaldehyde and counterstained using DAPI (Thermo Fisher D1306). An EVOS FL fluorescence microscope was used to acquire 20X images of the porous membrane bottom at five random locations for each sample^50^. The number of invading cells was manually counted for each technical replicate for n ≥ 4 biological replicates.

### Lentiviral transfection and *in vivo* tumor model

All animal procedures were approved by the Institutional Animal Care and Use Committees (IACUC) at the University of Virginia and Virginia Polytechnic Institute and State University. Lentivirus conferring expression of green fluorescent protein (GFP) under puromycin antibiotic selection was a generous gift from the laboratory of Dr. Kevin Janes. Murine GL261 were serially transfected with GFP lentivirus and purified by selection with 2 μg/mL puromycin (Thermo Fisher A1113803). For *in vivo* tumor studies, a burr hole was drilled into the skull of anesthetized C57BL/6 mice (5–8 weeks; Harlan Laboratories) at coordinates −2, +2, −2.2 (AP, ML, DV) from bregma. 100,000 GFP^+^ GL261 cells were inoculated in 5 μL at 1 μL/min, and the bur hole was sealed with bone wax. Ketoprofen was administered at 2 mg/kg for 48 hours to manage pain. One week later, the inoculation site was re-exposed, and a blunt-end 26 gauge needle was used to infuse 10 μL of 1 mg/mL biotinylated dextran amine at 1 μL/min. Ketoprofen was again administered at 2 mg/kg for 48 hours to manage pain.

### Flow cytometry

Triplicate wells of 100,000 GL261 cells were cultured in serum-containing medium overnight, either on 2D tissue culture plastic or in 3D hydrogels, as described above. The following day, cells were cultured with 10 μM Brefeldin A in serum-free medium for 6 hours, harvested, pooled, and subjected to antibody labeling^7^. To assess expression *in vivo*, mice were inoculated with GFP + tumor cells as above, and 14 days post-implantation mice were treated with 0.25 mg Brefeldin A for 6 hours via intraperitoneal injection^51^. The brains were then dissociated for analysis. Briefly, the ipsilateral cortical hemisphere was isolated into HBSS and slightly trimmed to reduce the number of non-cancerous cells. The tissue was minced using a scalpel blade, incubated in 5 mL of ACK RBC lysis buffer for 3–5 minutes at room temperature, and centrifuged at 1100 rpm for 5 minutes. An approximately equal volume of 1.5 mg/mL Liberase DL (Sigma 5466202001) was then added to digest the tissue for 30 minutes on a rocker at 37 °C, pipetting up and down to ensure complete digestion.

The tissue slurry was then strained through a 40 µm cell strainer followed by 35 mL of HBSS. This solution was centrifuged at 1100 rpm for 5 minutes, and the isolated cells were resuspended and counted for flow cytometry. Primary-conjugated antibodies were used to stain for CXCR4 (eBiosciences 17-9991-80) and CXCL12 (R&D IC350C), along with appropriate isotype controls. Dead cells were stained using LIVE/DEAD® Fixable Green Dead cell stain kit (Thermo Fisher L23101). Stained samples were run on a Millipore Guava flow cytometer for a minimum of 50,000 events, and the data was analyzed using Incyte software. A flow chart of the gating strategy is shown in Supplemental Fig. 1. For data analysis, plots were gated based on data from single stained controls. *In vivo* samples were further gated on GFP^+^ cells to assess only GL261. All numbers are shown as percent of live, single cells.

### Magnetic resonance imaging

Animals were anesthetized and placed in a 7 T Clinscan small animal MRI (Bruker/Siemens, Ettlingen, Germany) equipped with a 30-mm head coil. A T2-weighted image was taken through the head with the following parameters: repetition time (TR) = 5500 ms, echo time (TE) = 65 ms, field of view (FOV) = 20 mm × 20 mm with a 192 × 192 matrix, slice thickness = 0.5 mm, number of slices = 30, two averages per phase-encode step requiring a total acquisition time of about 5 min per mouse. For T1-weighted MRI, a 33-Gauge, blunt-end catheter was placed into the same coordinates for tumor implantation, and 10 μL of 25 mg/mL Glowing Galbumin (BioPAL Inc.) was infused at a rate of 1 μL/min. Following an initial image, T1 images were acquired approximately 30 minutes, 1 hour, and 24 hours post-infusion according to the following parameters: TR = 500 ms, TE = 11 ms, FOV = 20 mm × 20 mm with a 192 × 192 matrix, slice thickness = 0.7 mm, number of slices = 22, two averages per phase-encode step requiring a total acquisition time of about 3 min per mouse. Contrast-enhanced T1-weighted images at time t = 0 were subtracted from images at t = 30 minutes to generate a difference heat map and visualize changes in contrast intensity over time.

### Tissue harvest and immunohistochemistry

Two days after convection enhanced delivery, tumor-bearing mice were administered Euthasol solution and intracardially perfused with phosphate buffered saline (PBS). Brain tissue was quickly harvested and bisected coronally at the center of the injection site. The brains were fixed overnight in 4% paraformaldehyde, cryopreserved in 30% sucrose, and sectioned at 12 µm using a Leica 1950 cryostat. Tissue sections were blocked in 3% serum and 0.03% Triton X-100 in PBS for 1 hour, then were incubated overnight at 4 °C with rabbit anti-pCXCR4 (Abcam ab74012) diluted in blocker buffer. The samples were washed three times with PBS and incubated for 1 hour at room temperature with goat anti-rabbit 660 diluted in blocking buffer. After washing again, the nuclei were counterstained using DAPI (Thermo Fisher).

### *In vivo* invasion quantification

Fluorescently labeled sections were imaged using an EVOS FL microscope. Five images were randomly taken around the tumor periphery for each of three sections 120 μm apart for each animal. The tumor border was identified based on GFP^+^ GL261 and nuclear staining, and a blinded investigator counted the number of GFP^+^ tumor cells beyond the border for each image. Data are presented as the number of invading cells per mm^2^ of tissue.

### Patient sample collection and immunohistochemistry

All procedures involving human participants were performed in accordance with the ethical standards of the Institutional Review Board of the University of Virginia and the 1964 Helsinki declaration and its later amendments or comparable ethical standards. The tissue samples were initially banked with informed patient consent. The samples in this study were accessed through the University of Virginia Biorepository and Tissue Research Facility selected by a neuropathologist (Dr. James W. Mandell, UVA) based on a definitive diagnosis of GBM (astrocytoma, WHO grade IV) who had completed tumor resections at the University of Virginia between 2010 and 2013. Samples were de-identified and processed to identify ones containing primarily tumor bulk. Descriptive statistics and survival information for the patient cohort are presented in Table 1.

For quantitative analysis, we started with ten samples taken prior to therapy and seven taken after standard of care therapy. The eight micron sections were deparaffinized in xylene followed by four graded washed with an increasing ratio of ethanol:water to achieve rehydration. The samples were then subjected to boiling in citrate buffer for 30 minutes for antigen retrieval. The samples were blocked in 3% donkey serum and 0.03% Triton X-100 for 1 hr at room temperature, then primary antibodies against CXCR4 (Sigma GW21075) and CXCL12 (Abcam ab18919) were added overnight at 4 °C. The following day, the samples were washed three times in 1X PBS, treated with donkey anti-rabbit 488 and donkey anti-chicken 647 for 1 hr at room temperature, then washed again. Nuclei were counterstained using DAPI (Thermo D3571), and the samples were mounted in Fluoromount-G (SouthernBiotech) prior to coverslipping. Tissue samples were scanned at 10X using an EVOS-FL Auto2. Five regions were randomly selected from each sample. Thresholding was performed for each image in ImageJ prior to measuring the integrated densities, used to obtain an average integrated density for each sample. Grubb’s test was then used to identify statistical outliers. For both CXCR4 and CXCL12 analyses, one pre-therapy sample was identified as an outlier and was therefore excluded from each.

### Statistics

Analysis of Variance (ANOVA) was performed for comparisons of more than two groups, using a significance level of 0.05. If significance was identified within the dataset, t-tests were performed to determine significance between individual groups. Ratio paired t-tests were used to analyze all *in vitro* data; unpaired t-tests were used to compare *in vitro* data to *in vivo* flow cytometry data; and unpaired student’s t-tests were used to compare experimental groups for *in vivo* invasion and patient sample quantification. All graphed and reported descriptive statistics in the text are presented as mean ± standard error of the mean, unless otherwise stated. Inferential statistics are reported as “(degrees of freedom) = value, n per group, p value” so that effect size can be determined from our reported data.

## Electronic supplementary material

Supplemental Figures 1 and 2
