# Supplementary material for: Convective forces increase CXCR4-dependent glioblastoma cell invasion in GL261 murine model
Source: Sci Rep. 2018 Nov 19;8:17057. doi: 10.1038/s41598-018-35141-9 (PMC6242861; doi:10.1038/s41598-018-35141-9)
Supplement: Supplementary file 1 — Supplemental Figures 1 and 2 [file 41598_2018_35141_MOESM1_ESM.pdf]

Supplemental information

"Convective forces increase CXCR4-dependent glioblastoma cell invasion in GL261 murine model"

R. Chase Cornelison[1], Caroline E. Brennan[2], Kathryn M. Kingsmore[2], and Jennifer M. Munson[1]*

[1]Department of Biomedical Engineering and Mechanics, Virginia Polytechnic Institute and State University, Blacksburg, VA 24061 [2]Department of Biomedical Engineering, University of Virginia, Charlottesville, VA 22908

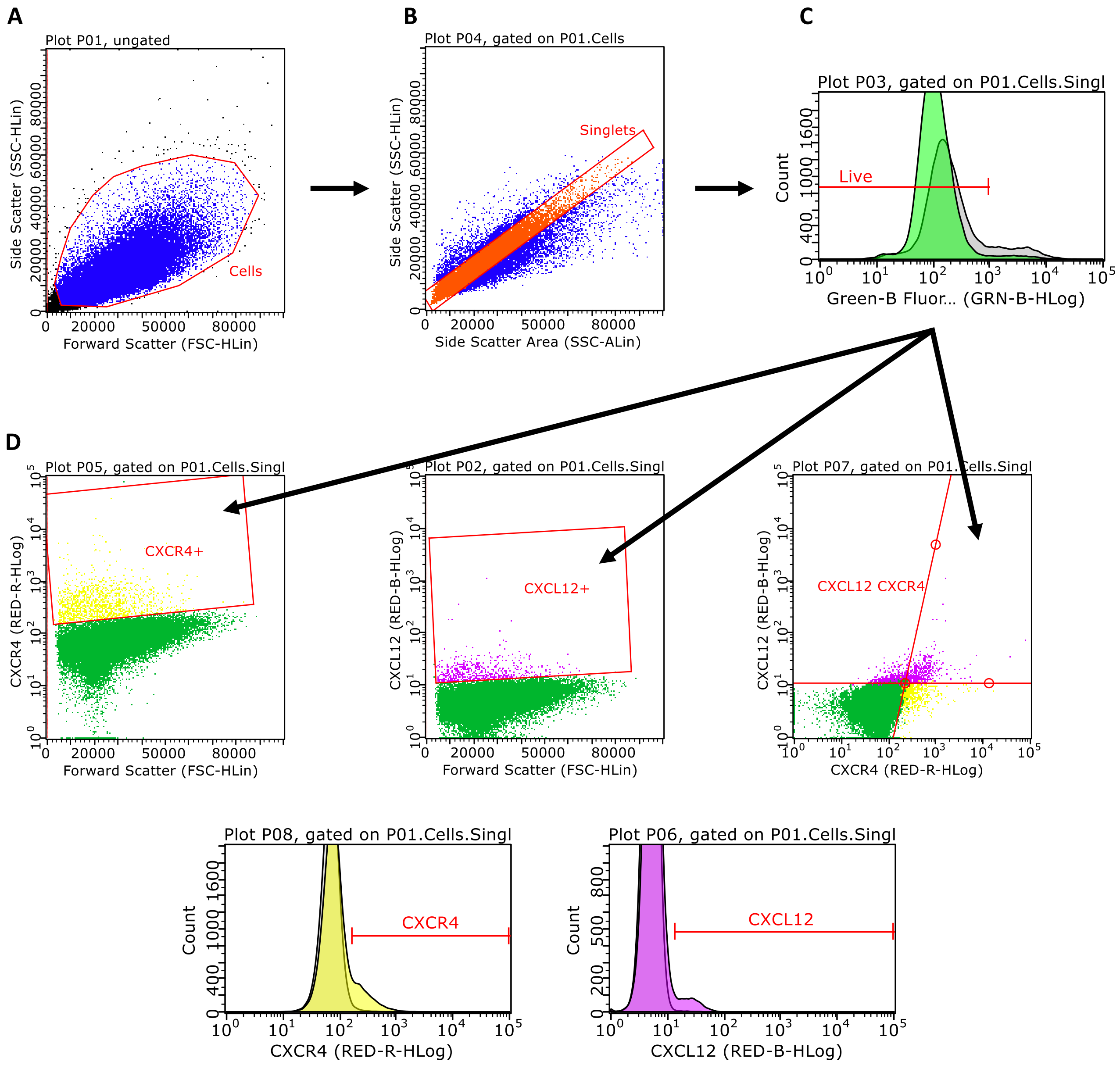

Supplemental Figure 1. Flow cytometry gating strategy. (A) A linear plot of side scatter versus forward scatter was used to gate on cells. (B) This gate was dragged to a linear plot of side scatter versus side scatter area, on which a rectangular gate was used to select only single cells opposed to doublets or triplets. (C) The Cells.Singlets gate was dragged onto the histogram for live/dead staining, after which a gate for live cells was drawn to include only the negative stained population. (D) This Cells.Singlets.Live gate used on all further plots and histograms for analysis.

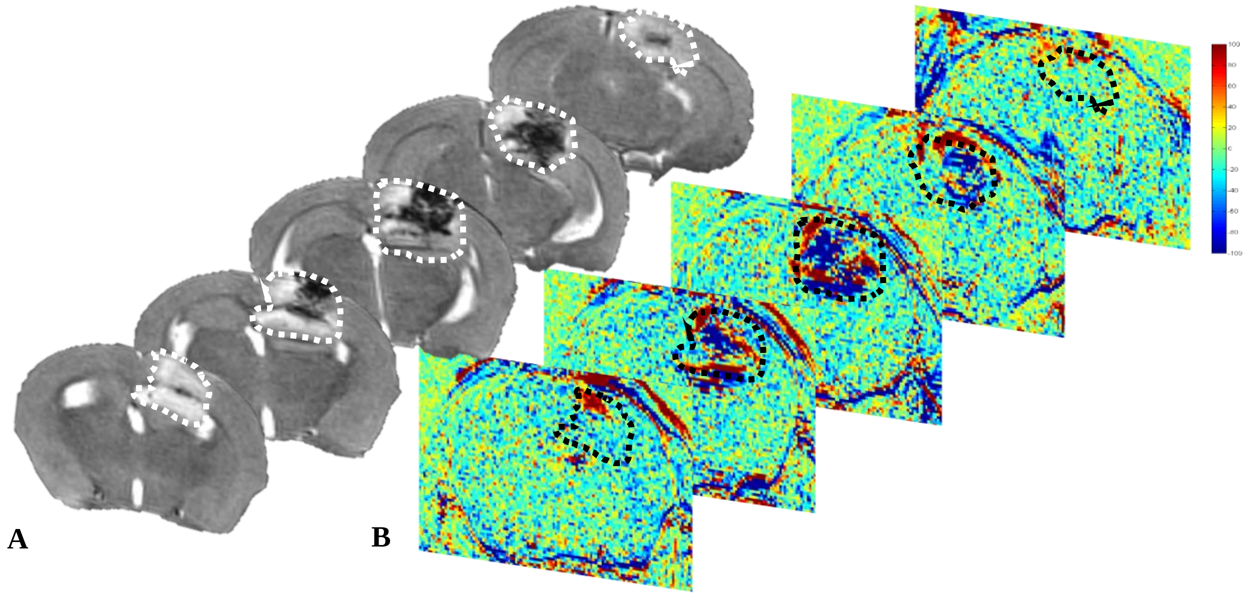

Supplemental Figure 2. Convective flow characterization using MRI. (A) T2-weighted images were used to identify tumor boundaries (outlined with white dashes) (B) Difference maps showing change in Galbumin contrast intensity over 30 minutes based on contrast enhanced T1-weighted images (tumor outlined with black dashes). Changes in contrast intensity during this time are indicated on a scale from blue to red (low to high).
